# The potential of Src inhibitors

**DOI:** 10.18632/aging.100821

**Published:** 2015-10-02

**Authors:** Daniel Elias, Henrik J. Ditzel

**Affiliations:** Department of Cancer and Inflammation Research, Institute of Molecular Medicine, University of Southern Denmark, Odense, Denmark

**Keywords:** Src family kinases, cancer

The src family of kinases (SFKs), including c-Src, Fyn, Yes, Lck, Lyn, Hck, Fgr and Blk, have been widely implicated in the pathogenesis of cancer [1]. Being pleiotropic, SFKs are involved in a number of different cellular activities such as regulation of cell growth, survival, adhesion, cytoskeletal remodeling and motility. Therefore, it is not surprising that aberrant activation of SFKs affects various aspects of tumor development and progression [2]. Consequently, substantial efforts have been made to develop drugs targeting these proteins, resulting in several FDA-approved drugs including imatinib, dasatinib and nilotinib, and a few in clinical trials such as saracatinib and bosutinib. While these SFK inhibitors have generally been well-tolerated with limited toxicity and good efficacy in hematological malignant diseases, efficacy in phase II clinical trials in different solid tumor indications have been modest. For example, multiple phase II clinical trials on dasatinib showed clinical benefit in less than a quarter of patients with breast cancer, prostate cancer or melanoma, and no measurable benefit in patients with small cell lung cancer or metastatic colorectal cancer; results with saracatinib and bosutinib were even more disappointing (reviewed in [3]. The reason for the failure of SFK inhibitors is unclear, but recent studies provide useful clues that might help in the design of future studies and subsequent improved clinical results.

One of the critical issues in the clinical development of SFK inhibitors is the lack of biomarkers to identify patients most likely to respond to such therapy. Our recent preclinical study using breast cancer cell line models showed that responsiveness to the SFK inhibitor PP2 depended on the level of expression or activation of SFKs [4]. This underscores the importance of identifying patients who have tumors with activated SFK signaling since they are more likely to benefit from SFK inhibitors. Second, SFK members may have opposing effects in different cancers. For example, a recent study in a breast cancer model demonstrated that knockdown of Fyn or Yes led to enhanced expression of Claudin-2, thereby reducing liver metastasis, whereas inhibition of Lyn (another member of the SFKs) resulted in reduced Claudin-2 expression with consequent increased liver metastasis [5]. This suggests that, due to the contradictory effects of some members of SFKs, the clinical efficacy of non-selective SFK inhibitors may be compromised, highlighting the need for the development of more specific agents.

It is clear that the tumor microenvironment (TME), which includes the cancer cells, blood vessels, the extracellular matrix (ECM), stromal cells, fibroblasts, immune cells, periocytes and adiposites, has a major impact on cancer pathogenesis. The immune cells in the TME include T and B lymphocytes, natural killer cells and tumor-associated macrophages (TAM). In many solid tumors, the presence of immune system cells favoring strong cell-mediated immune responses, such as CD8+ T cells and CD4+ T helper1 cells, is associated with good prognosis. On the other hand, the infiltration of tumors with TAM or B cells leads to pro-tumorigenic microenvironment, resulting in increased tumor burden and a consequent poor prognosis [6]. The SFKs play crucial roles in the development of host immune responses: Development and activation of T lymphocytes, natural killer cells, macrophages and dendritic cells is enhanced by increased expression or activation of SFKs (reviewed in [2]). It is therefore plausible to suggest that the use of non-specific SFK inhibitors in the treatment of malignancies may come at a price of inhibiting host immune resistance against tumor cells, and may even render patients vulnerable to infections. Indeed, a study focused on treatment of chronic myelogenic leukemia (CML) with dasatinib (SFK inhibitor) showed that these patients developed marked immunosuppression, involving T lymphocytes and NK cells [7]. Such adverse SFK inhibitor-mediated suppression of the host immunity again underlines the need for specific SFK inhibitors.

While SFK inhibitors administered in combination with other agents may show that these drugs have synergistic effects, a recent study showed that sequential treatment involving chemotherapy followed by SFK inhibitors lead to improved efficacy [8]. In a recent report Goldman et al showed that simultaneous administration of SFK inhibitor and taxane is less effective than sequential treatment where SFK inhibitor was administered following taxane therapy. This improved outcome may result from chemotherapy-induced adaptive phenotypic changes in cancer cells rendering them more vulnerable to treatment with SFK inhibitors [8]. This method, if consistently reproduced, may be a useful translational strategy for the management of cancer using SFK inhibitors.
